# 2527. Impact of Hypoalbuminemia on Clinical Outcomes Among Patients with Obesity Treated with Ceftriaxone

**DOI:** 10.1093/ofid/ofad500.2145

**Published:** 2023-11-27

**Authors:** Christina G Rivera (O'Connor), Omar M Abu Saleh, Jack W McHugh, Evan Draper, Kristin Mara, Kellie Arensman Hannan

**Affiliations:** Mayo Clinic, Rochester, Minnesota; Mayo Clinic Rochester, Rochester, Minnesota; Mayo Clinic, Rochester, Minnesota; Mayo Clinic, Rochester, Minnesota; Mayo Clinic, Rochester, Minnesota; Mayo Clinic Health System, Mankato, Minnesota

## Abstract

**Background:**

Due to extensive protein binding, use of ceftriaxone in the setting of hypoalbuminemia may result in suboptimal drug exposure via higher concentrations of unbound drug resulting in increased volume of distribution and increased drug clearance. We aimed to evaluate the impact of hypoalbuminemia on clinical success among hospitalized adults with obesity who were treated with ceftriaxone for a bacterial infection.

**Methods:**

This retrospective review included adult inpatients with weight > 100 kg or body mass index (BMI) > 40 who received ceftriaxone 2 grams intravenously every 12 hours for at least 72 hours for one of the following indications: bloodstream infection, pneumonia, urinary tract infection, non-enterococcal endocarditis, skin and soft tissue infection, or bone and joint infection. The primary outcome was clinical success, a composite of clinical cure and microbiologic cure. Clinical cure was defined as resolution of signs and symptoms compatible with active infection without need to change or extend antibiotic therapy due to inadequate effect of ceftriaxone. Microbiologic cure was defined as negative repeat sterile site cultures, if applicable. Secondary outcomes included length of stay, mortality, and 30-day readmission. Outcomes were analyzed with Mann-Whitney U tests, Pearson’s chi-squared tests, and Fisher’s exact tests (α level = 0.05).

**Results:**

137 patients were included, 34 of whom had a serum albumin ≤ 2.5 g/dL. Patients were predominantly white (94.2%) and male (69.3%) with a median BMI of 48.6 (Table 1). Clinical success, a composite of clinical and microbiologic cure, was achieved in 76.5% and 92.2% (*p* = 0.013) of those with and without hypoalbuminemia, respectively. Clinical cure was significantly more common among those without hypoalbuminemia (93.2%) as compared to those with hypoalbuminemia (76.5%) (*p* = 0.007). Death during hospitalization was more common (14.7% vs. 0%, *p* < 0.001) and median length of stay was longer (14 vs. 9 days, *p* = 0.009) in the hypoalbuminemia group. No difference was seen in 30-day readmission (23.5% vs. 11.7%, *p* = 0.089).

**Figure:**
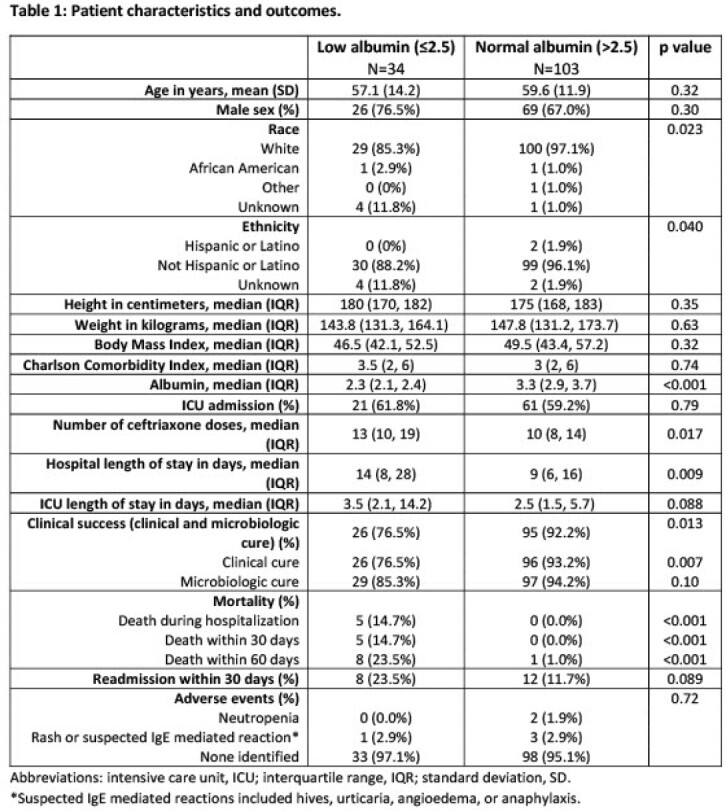

**Conclusion:**

Hypoalbuminemia was associated with a lower rate of clinical success among patients with obesity who were treated with ceftriaxone 2 grams every 12 hours.

**Disclosures:**

**Christina G. Rivera (O'Connor), Pharm.D**, Gilead Sciences: Advisor/Consultant|Gilead Sciences: Board Member|Gilead Sciences: Grant/Research Support|Gilead Sciences: Honoraria

